# Intrahippocampal Adeno-Associated Virus–Mediated Overexpression of Nerve Growth Factor Reverses 192IgG-Saporin–Induced Impairments of Hippocampal Plasticity and Behavior

**DOI:** 10.3389/fnins.2021.745050

**Published:** 2021-11-15

**Authors:** Yulia V. Dobryakova, Yulia S. Spivak, Maria I. Zaichenko, Alena A. Koryagina, Vladimir A. Markevich, Mikhail Yu. Stepanichev, Alexey P. Bolshakov

**Affiliations:** Institute of Higher Nervous Activity and Neurophysiology, Russian Academy of Science, Moscow, Russia

**Keywords:** nerve growth factor, 192IgG-saporin, behavior, long-term potentiation, septum, hippocampus, NGFR, TrkA

## Abstract

One of the aspects of Alzheimer disease is loss of cholinergic neurons in the basal forebrain, which leads to development of cognitive impairment. Here, we used a model of cholinergic deficit caused by immunotoxin 192IgG-saporin to study possible beneficial effects of adeno-associated virus (AAV)–mediated overexpression of nerve growth factor (NGF) in the hippocampus of rats with cholinergic deficit. Suspension of recombinant AAV carrying control cassette or cassette with NGF was injected into both hippocampi of control rats or rats with cholinergic deficit induced by intraseptal injection of 192IgG-saporin. Analysis of choline acetyltransferase (ChAT) immunostaining showed that NGF overexpression in the hippocampus did not prevent strong loss of ChAT-positive neurons in the septal area caused by the immunotoxin. Induction of cholinergic deficit in the hippocampus led to impairments in Y-maze and beam-walking test but did not affect behavioral indices in the T-maze, open field test, and inhibitory avoidance training. NGF overexpression in the rats with cholinergic deficit restored normal animal behavior in Y-maze and beam-walking test. Recording of field excitatory postsynaptic potentials *in vivo* in the hippocampal CA1 area showed that induction of cholinergic deficit decreased magnitude of long-term potentiation (LTP) and prevented a decrease in paired-pulse ratio after LTP induction, and NGF overexpression reversed these negative changes in hippocampal synaptic characteristics. The beneficial effect of NGF was not associated with compensatory changes in the number of cells that express NGF receptors TrkA and NGFR in the hippocampus and medial septal area. NGF overexpression also did not prevent a 192IgG-saporin–induced decrease in the activity of acetylcholine esterase in the hippocampus. We conclude that NGF overexpression in the hippocampus under conditions of cholinergic deficit induces beneficial effects which are not related to maintenance of cholinergic function.

## Introduction

The basal forebrain cholinergic neurons in the medial septal area, diagonal band of Broca (MS/DBB), and nucleus basalis innervate various structures of the brain. Their major functions include modulation of synaptic plasticity and excitability of cortical and hippocampal neurons. Loss of basal forebrain cholinergic neurons is typical of pathogenesis of several neurodegenerative diseases including Alzheimer disease and severe cases of Parkinson disease. The link between pathological manifestations and degeneration of cholinergic neurons is supported by findings made in animal studies where disruption of cholinergic signaling leads to impaired synaptic plasticity (Hirotsu et al., 1989; Sokolov and Kleschevnikov, 1995; Motooka et al., 2001; Dobryakova et al., 2020a), altered network functioning (Dannenberg et al., 2017), and cognitive deficits (Blokland, 1995). Furthermore, degeneration of cholinergic neurons by administration of saporin-based immunotoxins impairs learning and memory (Schliebs et al., 1996; Bolshakov et al., 2020). For instance, the immunotoxin induced motor deficit in the beam-walking test, hyperactivity, reduced T-maze alternation (Lehmann et al., 2002), and impaired behavioral performance in a Morris water-maze task (Walsh et al., 1995; Dobryakova et al., 2018). In contrast, cholinergic agonists can enhance cognition (Drachman and Leavitt, 1974), and potentiation of cholinergic function by acetylcholinesterase inhibitors improves cognitive state of patients with Parkinson disease (Emre et al., 2004; Pagano et al., 2015).

The alternative approach to treatment of cholinergic dysfunction is provision of trophic support to cholinergic neurons. It is known that nerve growth factor (NGF) is a neurotrophin critical for survival of cholinergic neurons in the adult basal forebrain (Williams et al., 1986; Hefti, 1994; Winkler and Thal, 1995). Under normal conditions, the major source of NGF for cholinergic neurons in the MS/DBB is the hippocampus, where NGF is produced by interneurons (Zeisel et al., 2015; Cembrowski et al., 2016). Cholinergic neurons express on their terminals in the hippocampus two NGF receptors, NGFR/p75 and NTRK1/TrkA; after binding to these receptors, NGF is transported along axons from the hippocampus to the soma of cholinergic neurons in the medial septal area. Loss of septohippocampal connection after fimbria/fornix lesion was shown to result in death of septal cholinergic neurons, which may be prevented by intracerebroventricular injection of NGF or its overexpression in the medial septal area (Williams et al., 1986; Blesch et al., 2005).

The positive effect of NGF on the animals with cholinergic hypofunction was described in several previous studies (Fischer et al., 1991; Bishop et al., 2008; Conner et al., 2009; Zhang et al., 2013; Dobryakova et al., 2020a), and it is likely to be related to recovery of cholinergic innervation of the hippocampus (Eu et al., 2021). However, the majority of these studies were predominantly focused on the elucidation of potential beneficial effect of NGF on behavior of animals with model pathology. The mechanisms behind positive effects of NGF remain poorly studied. The predominant idea is that NGF binds with the aforementioned receptors and produces positive effect via modulation of functioning of cholinergic fibers (Fischer et al., 1991; Cooper et al., 2001; Zhang et al., 2013). Our previous study using lentivirus construction focused on the behavioral changes showed that NGF overexpression in the hippocampus may only partly protect against behavioral disturbances caused by cholinergic deficit, and complete compensation may require more complex treatment (Dobryakova et al., 2021). However, several important questions remained unanswered, including what was the real level of NGF overproduction and whether this overproduction did modify the expression of NGF receptors in the brain. Finally, whether the depletion of ACh supply in the hippocampus would affect synaptic plasticity and would NGF overexpression in rats with cholinergic depletion be associated with changes in synaptic plasticity. Therefore, this study was designed to tackle these questions.

## Materials and Methods

Experiments were performed with adult male Wistar rats (250–350 g) received from the Nursery for Laboratory Animals, Branch of the Institute of Bioorganic Chemistry RAS (Pushchino). All procedures were approved by the ethical principles stated in the Directive 2010/63/EU of the European Parliament and of the Council of 22 September 2010 and were approved by the Ethical Committee of the Institute of Higher Nervous Activity and Neurophysiology of the Russian Academy of Sciences.

Animals belonged to one of three groups: 192IgG-saporin–injected rats with control virus expressing green fluorescent protein (GFP) (SAP-GFP; *n* = 10 for electrophysiological experiments, *n* = 9 for behavioral experiments), phosphate-buffered saline (PBS) injected control rats treated with control virus construction (PBS-GFP; *n* = 8 for electrophysiology, *n* = 10 for behavior), and 192IgG-saporin–injected rats treated with adeno-associated virus (AAV) carrying cassette with NGF and GFP separated by internal ribosome entry site (IRES) (NGF-IRES-GFP) (SAP-NGF; *n* = 9 for electrophysiological experiments, *n* = 10 for behavioral experiments). A total of 56 rats were involved in the study. During the experiments, all animals were maintained with a 12-h light–dark cycle and had *ad libitum* access to food and water.

### Plasmids Synthesis and Virus Preparation

Shortly, HEK293TN cells were grown to 70–80% confluence in Dulbecco modified eagle medium supplemented with 5% fetal bovine serum. Cells were cotransfected with pD2 (serotype 2) + pAAV-CAG-NGF-IRES-GFP (vector carrying human NGF cDNA) or pD2 + pAAV-CAG-IRES-GFP (control viruses) using branched PEI (Sigma). Three days after transfection, the cells were collected and lysed in a cell lysis buffer (3 × freezing/thaw at −80°C); cell debris were removed by centrifugation (3,900 g, 5 min, 4°C). Virus suspension was treated with Benzonase nuclease and incubated in water bath at 37°C for 30 min; then, the viral suspension was centrifuged (3,900 g, 5 min, 4°C), and supernatant was filtered (PES 0.45 μm). AAVs were purified using HiTrap HEPARIN HP columns and concentrated on Amicon 100 k filters. Then the viruses were sterilized using an Acrodisk filter (0.22 μm) and stored at −80°C. Viral titer was 10^11^–10^12^ viral particles/mL.

### Stereotaxic Surgery and Drug Administration

Drug administration was performed using standard stereotaxic methods. Animals were anesthetized with isoflurane (Baxter, United States) and mounted in a Kopf stereotaxic frame. 192IgG-saporin (1.5 mg) or PBS was injected into the medial septum area (0.4 mm anterior, 1.5 lateral to bregma, angle 14°). This dose of the toxin was chosen based on our previous data that the immunotoxin dose of 1.5 mg induced loss of choline acetyltransferase (ChAT)–positive neurons in the medial septum area (Dobryakova et al., 2020b, a). AAV suspension was bilaterally injected into the CA1 area of the hippocampus (2.9 mm posterior, 1.7 lateral to bregma, 3.3 mm depth; 1 μL/side). All injections were performed through the Hamilton syringe (Hamilton company, United States) using a microinfusion pump (Stoelting Co., United States) at a rate of 0.5 μL/min. After each injection, the needle was left *in situ* for 10 min. Rats were allowed to recover for 21 days after the surgery.

### Electrophysiology

Electrophysiological experiments were performed as previously described (Dobryakova et al., 2020a). Rats were anaesthetized with urethane (1.75 g/kg, intraperitoneally) and mounted in a stereotaxic frame for surgical preparation for the recording session. Stimulating nickel-chrome electrode (diameter 80 μm) was implanted into the ventral hippocampal commissure (VHC) (1.3 mm posterior, 1.0 mm lateral to bregma, approximately 3.5 mm ventral to dura). Recording electrode was placed into the CA1 area (2.7 mm posterior, 1.5 lateral to bregma, approximately 2.2 mm ventral to dura) (Paxinos and Watson, 2007). One electrode under the skin served as a ground and as a reference electrode.

To study whether NGF overexpression can prevent disturbances caused by the immunotoxin, the fEPSP amplitude in the CA1 field evoked by paired VHC stimulation (interstimulus interval 30 ms; intertrain time 20 s at intensity of 100–300 μA; 10 paired stimulations) was recorded every 10 min. The intensity of testing paired pulse stimulation was set to evoke 40–50% of the maximum fEPSP amplitude. The magnitude of stimulation current for paired pulse stimulation did not differ between the groups [138 ± 14, 145 ± 6, and 133 ± 11 μA for PBS-GFP, SAP-GFP, and SAP-NGF, respectively; *F*(2, 24) = 3.95, *p* = 0.68]. The amplitude of baseline fEPSP also did not differ between the groups [0.99 ± 0.09, 1.0 ± 0.13, and 1.0 ± 0.1 mV for PBS-GFP, SAP-GFP, and SAP-NGF, respectively; *F*(2, 24) = 0.02, *p* = 0.98]. Long-term potentiation (LTP) in the CA1 was induced by high-frequency stimulation of VHC (theta burst stimulation, five series, four trains of five stimuli with frequency of 100 Hz, intertrain interval was 200 ms; interseries interval was 30 s) after 30-min baseline recording. In our experiments for long-term recordings, we applied urethane anesthesia, which is used for non-recovery procedures of exceptionally long duration where preservation of autonomic reflexes is essential and thus does not need any additional euthanasia procedure.

### Behavioral Studies

#### T-Maze Spontaneous Alternation (Days 21–25)

The T-maze consisted of start alley (50 × 16 cm) and two goal arms (50 × 10 cm). It is equipped with central partition between goal arms and was surrounded with a wall (height 32 cm). The spontaneous alternation test was made in accordance with Deacon and Rawlins’ protocol (Deacon and Rawlins, 2006). For the first trial, a rat was placed at a starting point, and all guillotine doors were raised. When the rat chose the goal arm, the door was slid down. After 30 s, the door was opened, and the rat was placed in the start area of the central partition and allowed to choose between goal arms. Testing procedure lasted 5 days and involved 10 trials for no more than 2 min each.

#### Open Field (Day 26)

Exploratory activity of the rats was recorded in the round open field as described in the previous study (Dobryakova et al., 2018). The arena was divided in three equal concentric zones (central, mid, and peripheral). Each rat was placed in the center of the arena, and the rat behavior was recorded automatically for 5 min under red light using Ethovision software (Noldus, Wageningen, Netherlands). The following behavioral variables were quantified: total distance moved (cm), velocity (cm/s), movement frequency, total movement duration (s), vertical activity (rearings), grooming, and number of entries to the center of the arena.

#### Beam Walking (Days 27–31)

The sensorimotor coordination was assessed in a beam-walking test following the protocol described by Lehmann et al. (2000, 2002). The test apparatus consisted of a plastic beam (200 cm) elevated 80 cm above the floor and divided into four 50-cm segments and was connected to the goal box. During the first day session, rat was placed on the beam at 50 cm from the box at five sequential trials. On the next day, the rat was placed at a distance of 50, 100, 150, and 200 cm from the goal box, consequently, with only one trial for each distance. The third session included only two trials with 100- and 200-cm distances. During the fourth session day, three sequential trials with 200-cm distance were made. On the fifth day, the rats were tested as in the fourth session, and their learning scores were recorded and estimated. The beam was divided into four segments. The maximal score was the sum of scores of three trials (maximum score 12). The score of 0 was given if the rats slipped down their paws or toes from the beam surface; the score of 1 was given if the rats passed the segment without slipping.

#### Y Maze (Day 33)

Spatial working memory and spontaneous alternation were studied in the Y-maze as previously described (Stepanichev et al., 2016). Test apparatus consisted of three equal plastic arms (120°; 42.5 × 14.5 × 22.5 cm). For the trial, a rat was placed at a starting point in the end of one of the arms facing the wall. The test is used to assess spontaneous alternation behavior, which is based on the ability of rats to remember the arm they have just explored and not enter in the previous choices. Testing procedure involved one trial for 8 min, during which they could explore freely all the arms of the maze. The series of arm entries were recorded. An alternation was defined as subsequent entries into all three arms. The maximum number of alternations was therefore the total number of entries minus two. The percentage of alternation was assessed as (alternation/maximum alternation) × 100.

#### Passive Avoidance Training (Days 33–34)

The test apparatus (Harvard Apparatus) consisted of a plastic box divided into two equal compartments (30 × 30 × 30 cm): one was brightly illuminated, and the other was dark. The floor of both compartments was made of stainless grid (0.5-cm diameter) separated by a distance of 1 cm. Intermittent electric shocks (50 Hz, 5 s, intensity of 1.5 mA) were delivered to the grid floor of the dark compartment by an isolated stimulator. The two compartments were separated by a door. During the first trial, rat was placed into the light compartment; 30 s later, the door between the compartments was opened, and the latency to enter the dark compartment with all four paws was recorded. The rat was given 60 s to cross the compartments border. Once the rat crossed with all four paws to the next compartment, the door was closed. After 30 s, the rat was removed from the apparatus. This habituation procedure was repeated three times. After each trial, rat was removed from the apparatus and returned into it in 30 min. On the third session, a 1.5-mA foot shock was administered for 5 s. In 24 h, during the retention trial, no foot shock was given, and the step-through latency was recorded as a measure of retention. The experiment was carried out similarly to the acquisition trial, except that the guillotine door did not close when the rat entered the dark compartment, and the shock was not applied to the grid floor. If the animal remained in a light compartment and did not cross within 300 s to the dark compartment, the session was ended.

### Brain Tissue Collection

All rats were anesthetized with urethane (1.75 g/kg) and were perfused transcardially with ice-cold 0.9% NaCl. The brains were removed, and the frontal part of the brain, which contains septum, was dissected and postfixed in 4% formalin (Panreac, Spain) solution in PBS (Biolot, Russia) for at least 2 days. The rest of the brain was cut along the midline. The hippocampus was isolated from the left hemisphere and used for assessment of NGF content and AChE activity. The right hemisphere was postfixed in the 4% formalin for control of transduction efficiency and immunohistochemistry.

### Peroxidase-Based Immunohistochemistry and Immunofluorescence

The full extent of septum and right hemisphere of the hippocampus were sectioned at a 50-μm-thick coronal brain slices using a vibrating blade microtome (VT1200 S; Leica, Germany). Two slices from the septum area of each rat were selected and stained for ChAT to confirm cholinergic depletion in the medial septum area. Two slices from the hippocampal area from each rat were selected and stained for GFP to confirm the virus expression. To study the distribution of p75/NGFR receptors in the hippocampus and medial septal area, several slices from these structures from each experimental group were double-stained for p75/NGFR and NeuN. Slices from the hippocampus and medial septum area were also stained for TrkA to determine the localization of the receptor after NGF overexpression.

For ChAT immunostaining, the sections were preincubated in 0.3% triton X-100 (SERVA, Germany) in 0.01 M PBS (PBS-T) three times for 5 min at room temperature. Endogenous peroxidases were quenched using 15-min incubation in 3% H_2_O_2_, and the sections were washed thrice with PBS-T. Then, the sections were incubated for 1 h in blocking solution [5% normal goat serum or normal rabbit serum (Sigma–Aldrich, United States) in PBS-T] and after that in blocking solution with primary rabbit anti-choactase antibodies diluted as 1:500 (Santa Cruz Biotechnology, United States) or goat anti-TrkA antibodies diluted as 1:20 (R&D Systems, United States) at 4°C overnight. The next day, the sections were washed in PBS-T and incubated with secondary antibodies [1:800, goat anti-rabbit immunoglobulin G (IgG)–biotin, Sigma–Aldrich or 1:500 rabbit anti-goat IgG-biotin, Jackson Laboratories, both United States] in blocking solution at room temperature for 1 h. After additional washing in PBS, the sections were incubated with avidin–biotin–horseradish peroxidase (HRP) complex (ABC Elite kit, Vector Labs, United States) for 1 h, and 3,3-diaminobenzidine (Sigma–Aldrich) was used as a chromogen for development of staining.

GFP fluorescence was enhanced using immunostaining. In brief, after the preincubation step as above, the sections were placed for 1 h in blocking solution (5% normal goat serum in PBS, Sigma–Aldrich) and after that in blocking solution with primary anti-GFP antibodies diluted as 1:500 (Invitrogen, United States) at 4°C overnight. The next day, the sections were washed in PBS-T and incubated with secondary goat anti-rabbit Alexa 488 conjugated antibodies (diluted as 1:500 in the same blocking solution at room temperature for 1 h).

### Double Peroxidase-Based Immunohistochemistry

Two additional sections from the hippocampus and medial septal area were double-stained for p75 and NeuN. In brief, the sections were washed in PBS three times for 5 min at room temperature. Endogenous peroxidases were quenched using 15-min incubation in 3% H_2_O_2_, and the sections were additionally washed thrice with PBS. Then, they were incubated for 1 h in blocking solution [0.3% triton X-100 (SERVA, Germany)] in 0.01 M PBS (PBS-T) and 5% normal goat serum (Sigma–Aldrich) and, after that, in blocking solution with primary mouse monoclonal anti-p75 NGF receptor antibodies diluted as 1:500 (Abcam, United Kingdom) at 4°C overnight. The next day, sections were washed in PBS and incubated with goat anti-mouse IgG-biotin, antibodies 1:500 (Jackson Laboratories, United States) in blocking solution at room temperature for 2 h. After additional washing in PBS, the sections were incubated with avidin–biotin–HRP complex (ABC Elite kit, Vector Labs, United States) for 1 h, and 3,3-diaminobenzidine with nickel enhancement (Vector Labs, United States) was used as a chromogen for development of staining. Then, the brain sections were washed thrice with PBS for 5 min and then placed in blocking solution with rabbit anti-NeuN antibodies diluted as 1:500 (Chemicon, United States) at 4°C overnight. The next day, the sections were washed and incubated with avidin–biotin–HRP complex (ABC Elite kit, Vector Labs, United States) for 1 h, and chromogenic detection was performed using Vector NovaRed Substrate Kit (Vector Labs, United States).

### Imaging and Cell Counting

All images were acquired with an epifluorescent Leica DM6000B microscope (Leica). Imaging parameters were set to avoid signal saturations. Two sections from the medial septal area were selected in accordance to rat brain atlas (Paxinos and Watson, 2007) with a distance of 300 μm between them. All ChAT-positive cells within the medial septum area were counted. The mean number of cells per section was counted and was considered as a representative value for one animal. The density of cells, expressing p75 and TrkA receptors, was calculated in the adjacent sections, using eye peace grid and a × 40 lens. Number of cells is presented as percent of the value counted in the PBS-GFP group.

### Nerve Growth Factor Measurements

After the electrophysiological recording, animals were submitted to transcardial perfusion with ice-cold 0.9% NaCl. The isolated left hippocampus was used for NGF assessment. To extract proteins, the hippocampal samples were homogenized in PBS containing inhibitor cocktail (Roche, Switzerland) and 0.1% NP-40 (Helicon, Russia). Then, protein lysates were kept on ice for 15 min to allow complete tissue disintegration and cell lysis. This step was followed by sample centrifugation at 15,000 g for 30 min at 4°C. The supernatants were used to detect rat NGF concentration in the hippocampus using a rat β-NGF enzyme-linked immunosorbent assay (ELISA) kit RAB0883 (Sigma–Aldrich). Briefly, to perform the assay, 100-μL volumes of samples (duplicate) or standards were added to the wells, followed by 100-μL volume of the detection antibody. After 1-h incubation at room temperature on a plate shaker set to 300 revolutions/min (rpm), the wells were washed 3 × 300 μL 1X wash buffer to remove unbound material. Then, 100 μL of Streptavidin solution was added to each well and incubated for 45 min at room temperature. Then, TMB One-Step Substrate Reagent was added to each well followed by incubation for 30 min in the dark (avoiding standard signal saturation) on a plate shaker set to 300 rpm. This reaction was stopped by addition of 100 μL of Stop Solution to each well, completing any color change from blue to yellow. Signal was generated proportionally to the amount of bound analyte, and the intensity was measured at 450 nm with a plate reader.

### Measurements of AChE Activity

The activity of AChE was assessed using a standard method for continuous monitoring of AChE activity in a fluorescence microplate using the Amplex Red Assay Kit (Thermo Fisher Scientific). In the kit, AChE activity is monitored indirectly using Amplex Red reagent (10-acetyl-3,7-dihydroxyphenoxazine), a sensitive fluorogenic probe for H_2_O_2_. Isolated hippocampi were homogenized in ice-cold homogenization buffer consisting of 0.9% NaCl, 0.1% NP-40 (Helicon), leupeptin (Sigma–Aldrich), and aprotenin (Sigma–Aldrich). Then, protein lysates were kept on ice for 15 min to allow complete tissue disintegration and cell lyses. Homogenates were centrifuged at 15,000 *g* for 30 min at 4°C. The supernatants were used to detect acetylcholinesterase. All samples were diluted in 1*X* reaction buffer from the kit. A volume of 100 μL was used for each reaction. The samples and positive controls were duplicated. Then, 100 μL of the Amplex Red working solution was added to each well. Working solution contained 400 μM Amplex Red reagent, 2 U/mL HRP, 0.2 U/mL choline oxidase, and 100 μM acetylcholine. The fluorescence was measured at multiple time points for 30 min (6-min intervals) at 20°C. The excitation was measured in the range of 530–560 nm and emission detection at 590 nm. The results were expressed as U/g tissue weight. One unit of AChE is the enzyme that generates 1 μmol of TNB per min.

### Statistical Analysis

All data are presented as mean ± SEM. The distribution of variables in the samples was normal according to Shapiro-Wilk test. One-way analysis of variance (ANOVA) with a between-factor “Group,” followed by Tukey honestly significant difference *post hoc* test, was used to reveal the differences between the groups. Mixed-design ANOVA with “Group” and “Time after stimulation” as between and within factors, respectively, followed by Tukey HSD *post hoc* test, was used to analyze electrophysiological data. The differences were considered as significant at *p* < 0.05.

## Results

### Estimation of the Efficiency of Adeno-Associated Virus-Mediated Gene Transduction

**FIGURE 1:**
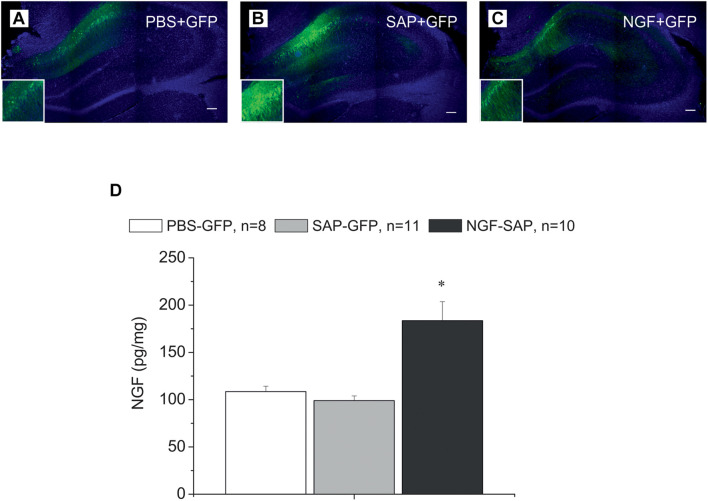
Immunostaining of hippocampal slices with antibody against GFP and DAPI staining in the hippocampus control rats **(A)** and rats with cholinergic deficit **(B,C)**, which were transduced with AAV carrying either GFP alone **(A,B)** or NGF-IRES-GFP **(C)**. The images of GFP staining (green) are superimposed over images of the same sections stained with DAPI (blue). **(D)** Shows NGF protein concentration determined by ELISA in the hippocampi of control (PBS-GFP, *n* = 8), 192IgG-saporin (SAP-GFP, *n* = 11), and NGF overexpressed rats (SAP-NGF, *n* = 10). Each point represents the mean ± SEM. ^∗^Significant differences compared to PBS-GFP and SAP-GFP groups, *p* < 0.05; bar, 200 μm.

Administration of AAV-based vectors in the hippocampus resulted in the thorough expression of carried genes in the CA1 and DG regions of the hippocampus (Figures 1A–C). Furthermore, in the NGF-SAP group, the level of NGF detected by ELISA in the hippocampal extracts was approximately twice higher as compared to the samples from PBS-GFP or SAP-GFP animals [Figure 1D; *F*(2, 26) = 13.5, *p* < 0.0001]. These data support the efficiency of neuronal transduction using our vectors.

### Nerve Growth Factor Overexpression Partially Improved Behavior of Rats Subjected to 192IgG-Saporin

**FIGURE 2:**
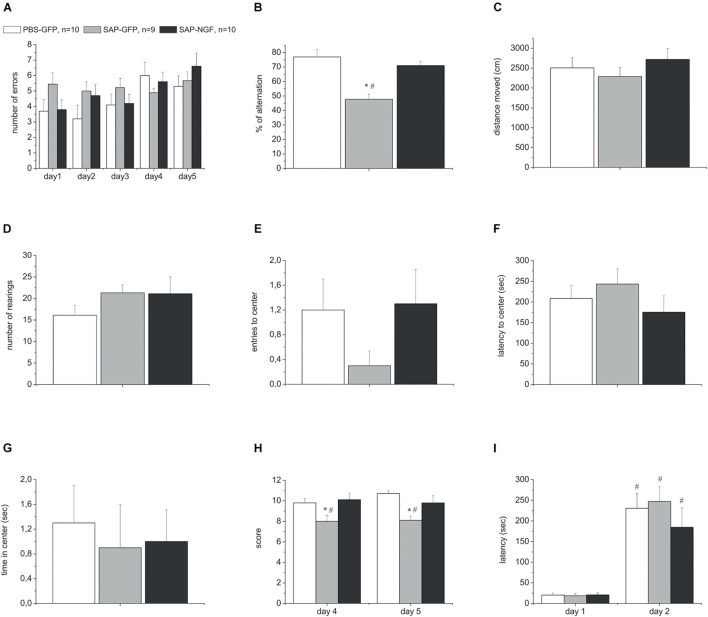
**(A)** Shows the number of errors over all testing days in the T-maze test. **(B)** Represents percentage of correct alternations in the Y-maze test. **(C–G)** Show distance moved, number of rearings, number of center crosses, latency to center, and time spent in the center in the open field test. **(H)** Shows motor beam-walking scores. **(I)** Represent the latencies during the first testing day and retention trial in the passive avoidance test. All rats were injected with PBS and control virus (PBS-GFP, *n* = 10), 192IgG-saporin (SAP-GFP, *n* = 9), and with 192IgG-saporin and NGF-carrying virus (SAP-NGF, *n* = 10). Each point represents the mean ± SEM. ^∗^Significant differences against the saline group, *p* < 0.05; ^#^significant intergroup differences, *p* < 0.05.

In order to estimate a possible effect of 192IgG-saporin and a possible protective effect of NGF after the simultaneous administration of the toxin and *Ngf*-containing or control vectors into the medial septum or hippocampus, respectively, we conducted a series of behavioral experiments. The series included several tests that allowed analyzing different aspects of animal behavior related to memory. Spontaneous alternation was assessed in the T-maze and Y-maze tests. These tests allow studying exploratory activity and short-term memory, although in a primitive form. In PBS-GFP rats, the overall number of errors over the first 3 days was lower compared to the SAP-GFP group in the T-maze test. NGF tended to decrease the number of errors. However, these changes were not significant (Figure 2A) (*p* = 0.05). The overall numbers of errors over all testing periods were not significantly different due to high number of errors over the last 2 testing days in all groups. In contrast, ANOVA revealed a significant group effect for the percentage of alternations [*F*(2, 26) = 14.5, *p* < 0.0001] in the Y-maze. 192IgG-saporin significantly decreased a percentage of correct choices and NGF overexpression induced a significant improvement of the alternation back to the control level (Figure 2B).

In order to conclude that the observed behavioral disturbances in the Y-maze were associated with effects on the mechanisms of short-term memory, and not on the general activity, rat locomotion and exploration were studied in the open field test. We did not reveal any significant differences in these indices among the groups injected with 192IgG-saporin, NGF or a combination of both (Figures 2C–G). We observed only a weak trend to a decrease in the number of center crosses in 192IgG-saporin–treated group (Figure 2E).

Additionally, the sensorimotor coordination was evaluated in the beam-walking test. Noteworthy, cholinergic deficit resulted in a significant reduction of the beam-walking score in SAP-GFP group (SAP-treated group vs. control group) whereas NGF overexpression in the hippocampus improved the sensorimotor coordination and, thus, increased the score in the SAP-NGF group compared to SAP-GFP rats. ANOVA also showed a significant group effect [*F*(2, 26) = 6.96, *p* < 0.01] for beam-walking scores (Figure 2H).

Finally, the behavior in a step-through passive avoidance task was used to evaluate the mechanism of long-term memory formation and recall. Twenty-four hours after the training session, all the animals spent most of testing time in the light part of the experimental box exhibiting normal memory capacities. Thus, neither induction of cholinergic deficit nor NGF overexpression influenced animal behavior in this test (Figure 2I). Altogether, our data indicate that intraseptal administration of 192IgG-saporin induced mild impairment of short-term memory and motor coordination without any significant effect on general locomotor and exploratory activity and long-term memory formation and/or recall.

#### Nerve Growth Factor Overexpression Did Not Protect Cholinergic Neurons From 192IgG-Saporin Toxicity

**FIGURE 3:**
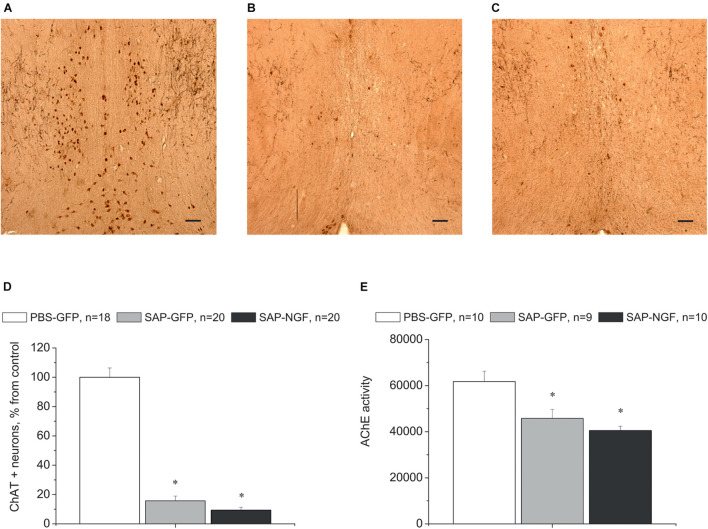
**(A–C)** Show immunostaining of ChAT in the medial septal area of rats treated with PBS **(A)**, 192IgG-saporin **(B)**, or 192IgG-saporin and NGF-carrying virus (SAP-NGF) **(C)**. **(D)** Shows the number of ChAT-positive neurons counted in the medial septum area. Bar, 200 μm. **(E)** Demonstrates acetylcholinesterase activity in rat hippocampi injected with PBS (PBS-GFP, *n* = 10), 192IgG-saporin (SAP-GFP, *n* = 9), and with 192IgG-saporin and NGF-carrying virus (SAP-NGF, *n* = 10) 1.5 month postinjection. Each point represents mean ± SEM. ^∗^Significant differences against the saline group, *p* < 0.05.

In order to assess to which extent these mild behavioral abnormalities were related to the capability of 192IgG-saporin to induce a loss of cholinergic neurons, we detected ChAT expression in the brain sections containing the medial septal area using specific antibodies. We found that intraseptal injections of the immunotoxin induced a loss of the majority of ChAT-positive neurons in the medial septal area. Depletion of ChAT-positive cells in this region ranged from 50 to 100% as compared to the control (Figures 3A–C). One-way ANOVA revealed a significant group effect for ChAT-positive cell count in the medial septal area [Figure 3D; *F*(2, 55) = 146.7, *p* < 0.01]. Noteworthy, the loss of cholinergic cells in the medial septal area was associated with a respective decrease in the AChE in the hippocampus. This effect was similar in both SAP-GFP and SAP-NGF groups studied (Figure 3E).

**FIGURE 4:**
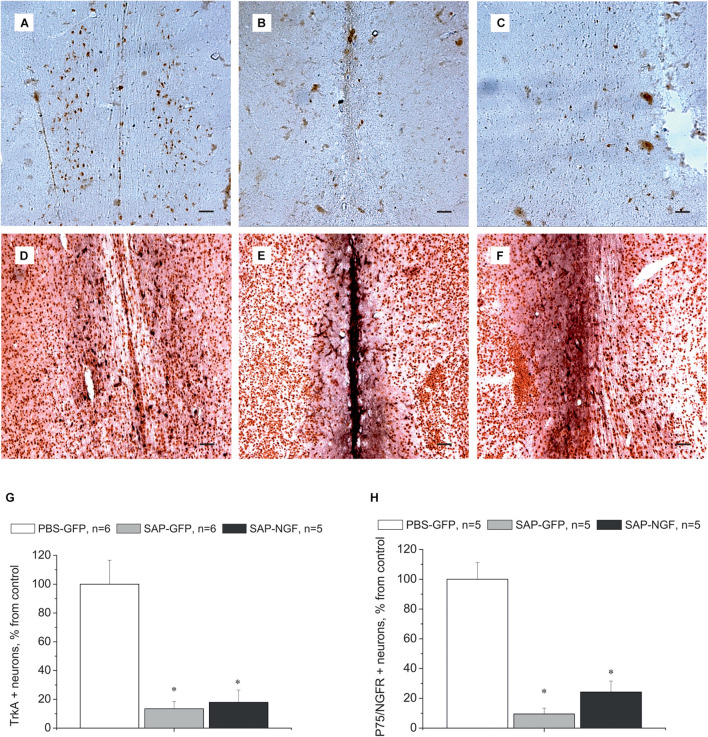
**(A–C)** Show medial septum regions from rats of the control group **(A)**, 192IgG-saporin–treated group **(B)**, and group treated with 192IgG-saporin and NGF-carrying virus (SAP-NGF) **(C)** stained with antibodies against TrkA. **(D–F)** Represent medial septum sections from PBS-treated **(D)**, 192IgG-saporin–treated, **(E)** and 192IgG-saporin–treated NGF-overexpressing rats **(F)** stained with antibodies against NeuN (brown) and p75/NGFR (black). **(G)** Shows the number of TrkA-positive neurons in the medial septum area. **(H)** Shows the number of p75/NGFR-positive neurons in the medial septum area. Bar is 400 μm. *Significant differences against the PBS-GFP group, *p* < 0.05.

Cholinergic neurons in the medial septum area express the TrkA and p75/NGFR receptors on the membranes of their processes and bodies to mediate NGF signal (Figures 4A,D). Administration of 192IgG-saporin into the septum led not only to a decrease in the number of ChAT-containing neurons but also to almost complete loss of TrkA [Figure 4B,G; one-way ANOVA *F*(2,14) = 9.0, *p* < 0.03] and p75 [Figures 4E,H; one-way ANOVA *F*(2, 12) = 36.5, *p* < 0.00001] staining in this area. Some TrkA-positive cell profiles (Figure 4C) and p75-positive processes (Figure 4F) could be observed in the brain of rats of the SAP-NGF group. These data additionally support the conclusion that 192IgG-saporin induced significant loss of cholinergic neurons in the medial septal area with very subtle, if any, action of NGF.

### Nerve Growth Factor Overexpression Significantly Improved Synaptic Plasticity in 192IgG-Saporin–Treated Rats

**FIGURE 5:**
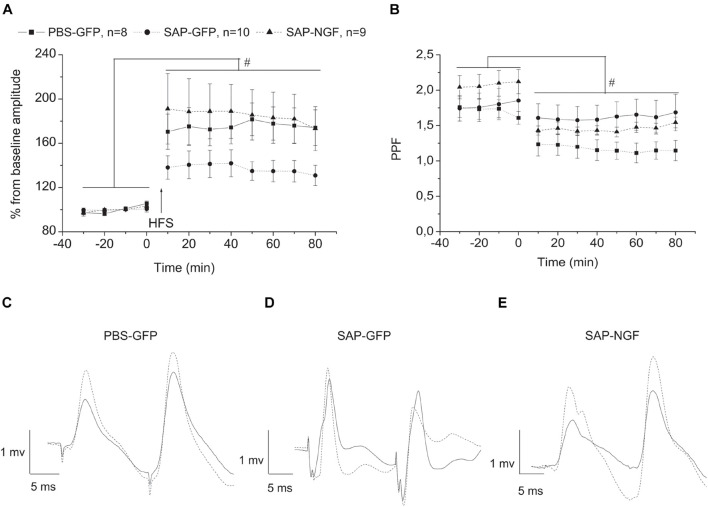
**(A)** Shows that cholinergic deficit induced by 192IgG-saporin significantly suppressed LTP induction in VHC-CA1 pathway [SAP-GFP (*n* = 10) and PBS-GFP groups (*n* = 8)]. NGF overexpression (SAP-NGF group, *n* = 9) facilitates the induction of hippocampal LTP as compared with SAP-GFP group. In **(B)**, the time course of PPF in PBS-GFP, SAP-GFP, and SAP-NGF groups. In **(C–E)**, dotted lines illustrate the fEPSP after HFS and solid lines, before HFS. Each point represents the mean ± SEM. Percentage of basal fEPSP amplitude at 0 min; #significant differences against the baseline, *p* < 0.05.

Could the mild effects of NGF overexpression on animal behavior be associated not with an improvement in cholinergic transmission but with an effect on neuroplasticity in the hippocampus? To tackle this question, we examined whether NGF overexpression under conditions of cholinergic deficit would have protective effect on the LTP induction at VHC-CA1 synapses *in vivo*. LTP-inducing tetanic stimulation was applied in animals when the baseline fEPSP was stable for at least 30 min. Analysis of fEPSP slope using mixed-design ANOVA revealed no significant group effect [*F*(2, 23) = 1.1, *p* < 0.3] and interaction between group × time [*F*(22, 253) = 1.0, *p* < 0.4]. However, significant time effect [*F*(11, 253) = 30.1, *p* < 0.00001] was found for this parameter reflecting LTP induction. In contrast, analysis of fEPSP amplitude revealed a trend to a group effect [*F*(2, 23) = 2.7, *p* < 0.09], a significant time effect [*F*(11, 253) = 34.8, *p* < 0.001], and strong time × group interaction [*F*(22, 253) = 2.3, *p* < 0.0001]. Data in Figure 5A demonstrate that although an increase in fEPSP amplitude was found in all groups of rats, the magnitude of this fEPSP increase in the SAP-GFP group was significantly lower as compared with the control or SAP-NGF rats. Thus, NGF overexpression prevented a negative effect of cholinergic deficit on the LTP magnitude. We also estimated paired-pulse facilitation (PPF) during baseline and after LTP induction (30-ms interstimulus interval) and did not observe any group effect on PPF whereas a strong effect of time [*F*(11, 253) = 31.94, *p* < 0.00001] and time × group interaction [*F*(22, 253) = 4.14, *p* < 0.0001] were found. The *post hoc* test revealed that LTP induction led to a significant decrease in the PPF under the control conditions (Figure 5B), which was absent in the SAP-GFP rats. The development of PPF after the LTP induction was normalized by NGF overexpression.

As field response is multicomponent and has contribution of different factors, we also evaluated changes that were produced by cholinergic deficit and NGF in the characteristics of second fEPSP. Unlike the characteristics of the first response, the second response was not affected by any of the treatments. Mixed-design ANOVA revealed no significant group effect [*F*(2, 21) = 0.18, *p* < 0.8] and interaction between group × time [*F*(20, 210) = 0.14, *p* < 1.0]. However, LTP was induced in all groups which, is supported by significant time effect [*F*(10, 210) = 30.1, *p* < 0.00001] for the amplitude of the second response. Similarly, the group effect was absent for the slope of the second response [*F*(2, 22) = 1.8, *p* < 0.19], and no interaction group × time interactions were observed [*F*(20, 220) = 1.3, *p* < 0.2]. However, significant time effect [*F*(10, 220) = 14.055, *p* < 0.0001] was also observed for the fEPSP slope reflecting LTP induction in all experimental groups. The data of electrophysiological experiments suggest that induction of cholinergic deficit leads to suppression of LTP magnitude and NGF restores it; however, this effect is observed only for the first fEPSP response.

### Nerve Growth Factor Overexpression Does Not Affect the Expression of TrkA and p75 Receptors in the Hippocampus

**FIGURE 6:**
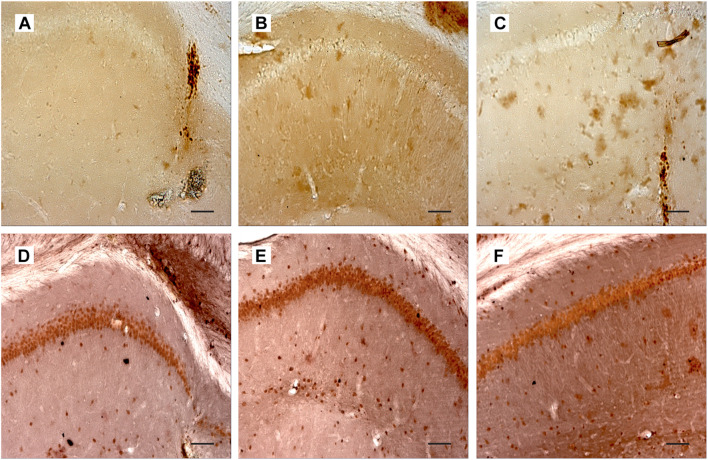
**(A–C)** Show hippocampal sections from PBS-treated **(A)**, 192IgG-saporin–treated **(B)**, and 192IgG-saporin–treated and NGF-overexpressing rats **(C)** stained with antibodies against TrkA. **(D–F)** illustrate hippocampal regions from rats treated with PBS **(D)**, 192IgG-saporin **(E)**, or 192IgG-saporin and NGF-carrying virus (SAP-NGF) **(F)** with antibodies against Neu (brown) and p75/NGFR (black). Bar is 200 μm.

We also studied whether the above effects were mediated by local overexpression of NGF receptors in the hippocampus. For this purpose, we detected expression of TrkA and p75 receptors in the hippocampus using specific antibodies. In the control rats, there were no neurons stained with anti-TrkA antibodies except of some cell profiles that were observed along the needle track (Figure 6A). Similar pattern of immunostaining was observed in two other groups of animals (Figures 6B,C). Single NGFR-positive cell profiles were scattered in the hippocampus, mainly in the region of injection, but the pattern of staining did not differ among groups (Figures 6D–F). Thus, overexpression of NGF produced in the hippocampus by AAV-mediated transduction did not substantially affect the expression of NGF receptors in this brain region.

## Discussion

In the present study, we injected male rats with 192IgG-saporin into the septum and AAV-NGF vector or respective control vector into the hippocampus. We expected that the toxin induces degeneration of cholinergic neurons, which send their processes to the hippocampus, and the increased secretion of NGF in the hippocampus will protect these neurons from the 192IgG-saporin–induced toxicity. It was mentioned above that NGF is a neurotrophin critical for survival of cholinergic neurons in the adult basal forebrain (Williams et al., 1986; Hefti, 1994; Winkler and Thal, 1995). That is why we hypothesized that the interaction of NGF with the receptors located on the processes of neurons from the medial septum should improve their survival. We also expected that this neuroprotection will improve behavioral impairments associated with 192IgG-saporin toxic action.

However, our hypothesis was supported only partially. It is well-known that cholinergic deficit results in some behavioral impairments (Nilsson et al., 1992; Lehmann et al., 2000, 2002; Gil-Bea et al., 2011) and does not influence other forms of behavior (Fletcher et al., 2007; Mchugh et al., 2015; Dobryakova et al., 2020b). In the present study, we also report that SAP-GFP rats treated with only 192IgG-saporin exhibited impaired short-term memory in a Y-maze and motor coordination during beam walking. NGF overexpression in the hippocampus of SAP-NGF animals improves behavior, which was impaired by induction of cholinergic dysfunction. These data are in accordance with our previous study, which was performed using rLV-based vectors to transduce neurons with the *Ngf* gene (Dobryakova et al., 2021). In the previous study, we used a model of cholinergic deficit induced by intracerebroventricular injection of 192IgG-saporin, which is associated with strong inflammation in the hippocampus (Dobryakova et al., 2019) and neocortex (Volobueva et al., 2020). We also observed beneficial effect of NGF in different behavioral tests, and here, we extend these findings using a model of cholinergic deficit, which is not associated with development of hippocampal inflammation. Importantly, in our previous study, we analyzed only behavioral deficits and possible protective effect of NGF without analysis of other parameters that may help to understand the mechanisms of the beneficial effect of NGF overexpression. Here, we confirm that NGF has beneficial behavioral effects and show that NGF also restores synaptic plasticity in the hippocampus. In addition, we showed that some improvement in behavior after NGF overexpression was not related to the attenuation of cholinergic deficit because the numbers of cholinergic neurons in the medial septal area and AChE activity in the hippocampus were similar to those found in the group of rats treated with 192IgG-saporin only.

What are the other mechanisms that may be responsible for the behavioral effects of NGF overexpression in 192IgG-saporin–treated rats? It is well-known that increased level of NGF in the hippocampus considerably facilitates synaptic plasticity and improves memory. Previous studies showed that NGF is responsible for neuron survival and promotes neurogenesis in the adult hippocampus (Frielingsdorf et al., 2007). In rats with cholinergic deficit induced by 192IgG-saporin, intracerebroventricular infusion of NGF or NT-3 rendered partial protection to ChAT-positive neurons (Lee et al., 2013). In contrast, a recent article demonstrated that AAV-mediated NGF overexpression in the mouse hippocampus did not change the level of cholinergic fiber depletion in the hippocampus compared to untreated wild-type mice (Eu et al., 2021). In accordance with this report, we observed no difference in the number of cholinergic neurons in the 192IgG-saporin–treated animals without or with NGF overexpression. Furthermore, the enhanced NGF production did not affect the expression of its receptors, such as TrkA and p75/NGFR, in the remaining cells, which project to the hippocampus. In the hippocampus, NGF overexpression did not induce any compensatory appearance of TrkA and p75/NGFR receptors in the place of injection or other regions either. These findings, along with previous report that NGF has only a limited capacity to enhance functioning of residual cholinergic neurons (Winkler et al., 2000), suggest that the positive effect of NGF is hardly related to recovery of cholinergic function in the hippocampus.

Our data demonstrate that 192IgG-saporin administration diminished synaptic plasticity in the LTP model. It is worth to note that classical LTP in the Schaffer collaterals-CA1 pyramids synapses does not lead to changes in the PPF (Schulz et al., 1995), and, in our study, we observed a strong decrease in the PPF after LTP induction. The latter suggests that the recorded field responses in our experiments *in vivo*, probably, had some inhibitory component, and LTP induction resulted not only in potentiation of excitation but also, presumably, suppression of inhibition during the first fEPSP, which led to disproportional potentiation of the first and second responses and change in PPF. Importantly, under conditions of cholinergic deficit, changes in PPF were absent showing a picture of classical LTP, which was observed in both first and second responses. Presumably, induction of cholinergic deficit removed cholinergic innervation critical for recruitment of hippocampal interneurons in the field response and therefore eliminated inhibitory component in the recorded responses. If our proposition is correct, then the most likely type of interneurons involved in the described effect have to be interneurons with depressing synapses, which provide strong inhibition during the first response and much weaker during the second activation. Presumably, this can be parvalbumin-positive or cholecystokinin-positive interneurons, which provide strong perisomatic inhibition in CA1 area and have depressing synapses (Palacios-Filardo et al., 2021). The proposed mechanism of changes in PPF during LTP induction suggests that possible cell targets of NGF overexpressed under conditions of cholinergic deficit may be interneurons. The effect of NGF therefore may be related not to recovery of LTP in synapses formed by Schaffer collateral on CA1 pyramids, because this LTP was preserved in all studied groups, but also to recovery of involvement of inhibitory interneurons in generation of field response. This recovery may result from enhancement of inhibition itself and/or enhancement of excitatory drive to inhibitory interneurons in CA1. Probably, these effects of NGF overexpression were beneficial for animal behavior. However, the molecular cascade behind the effect of NGF remains obscured because it is not mediated by changes in the expression of NGF receptors in the hippocampus. Presumably, NGF overexpression under conditions of cholinergic deficit may lead to activation of some alternative signaling pathways that are weakly active under normal conditions. For example, it was shown that NGF can interact with bradykinin receptor 2 (Petrella et al., 2020) and α9β1-integrin (Staniszewska et al., 2008). According to current data, the mRNA level of bradykinin receptor 2 in the hippocampus is absent (Cembrowski et al., 2016) or very low (Dobryakova et al., 2018), suggesting that it is hardly the target of NGF. The mRNA level of α9 and β1 integrin subunits is much higher at the level of hippocampal transcriptome (Dobryakova et al., 2018) and, according to the data of single-cell cortical transcriptome, both subunits may be present practically in all cell types (Tasic et al., 2018). Moreover, α9β1 integrin is a signaling receptor for NGF, which activates the MAPK (Erk1/2) pathway (Staniszewska et al., 2008), which is important not only for cell division, but also for synaptic plasticity (Sweatt, 2001). However, we cannot exclude the involvement of other pathways mediating the beneficial effect of NGF overproduction on neuroplasticity and cognitive functions in the animals with cholinergic deficit. Indeed, it is possible that the hippocampal overproduction of NGF may have some distant effects via improvement of interaction between the hippocampus and entorhinal cortex, which may be involved in the improvement of animal behavior in tests of mnemonic function (Loesche and Steward, 1977; Reeves and Smith, 1987; Ramirez et al., 1996, 2003).

## Conclusion

In conclusion, we now show that NGF driven by pD2-rAAV vector construct is capable of exerting neurotrophic effects, resulting in the recovery of alternation behavior, motor coordination, and hippocampal LTP impaired due to destruction of cholinergic circuitry, induced by intraseptal administration of 192IgG-saporin, in the adult mammalian brain. Given that the mechanism of this beneficial effect was not related to the protection of cholinergic neurons in the MS/DBB complex, future studies exploring the molecular machinery underlying this effect observed using gene delivery technology may be well worth pursuing.

## Data Availability Statement

The raw data supporting the conclusions of this article will be made available by the authors, without undue reservation.

## Ethics Statement

The animal study was reviewed and approved by the Ethics Committee of the Institute of Higher Nervous Activity and Neurophysiology of the Russian Academy of Sciences.

## Author Contributions

YD performed injections, performed and analyzed behavioral experiments and immunohistochemistry, performed AChE measurements, and wrote the manuscript. YS performed ELISA measurements and packed AAV. MZ performed behavioral experiments. AK packed AAV. VM acquired funding and designed the study. MS analyzed immunohistochemical data, designed the study, and wrote the manuscript. AB designed the study, performed fluorescent microscopy, and wrote the manuscript. All authors contributed to the article and approved the submitted version.

## Conflict of Interest

The authors declare that the research was conducted in the absence of any commercial or financial relationships that could be construed as a potential conflict of interest.

## Publisher’s Note

All claims expressed in this article are solely those of the authors and do not necessarily represent those of their affiliated organizations, or those of the publisher, the editors and the reviewers. Any product that may be evaluated in this article, or claim that may be made by its manufacturer, is not guaranteed or endorsed by the publisher.

## References

[B1] Bishop K. M. Hofer E. K. Mehta A. Ramirez A. Sun L. Tuszynski M. (2008). Therapeutic potential of CERE-110 (AAV2-NGF): Targeted, stable, and sustained NGF delivery and trophic activity on rodent basal forebrain cholinergic neurons. *Exp. Neurol.* 211 574–584. 10.1016/j.expneurol.2008.03.004 18439998 PMC2709503

[B2] Blesch A. Conner J. Pfeifer A. Gasmi M. Ramirez A. Britton W. (2005). Regulated lentiviral NGF gene transfer controls rescue of medial septal cholinergic neurons. *Mol. Ther.* 11 916–925. 10.1016/j.ymthe.2005.01.007 15922962

[B3] Blokland A. (1995). Acetylcholine: a neurotransmitter for learning and memory? *Brain Res. Rev.* 21 285–300. 10.1016/0165-0173(95)00016-X 8806017

[B4] Bolshakov A. P. Stepanichev M. Y. Dobryakova Y. V. Spivak Y. S. Markevich V. A. (2020). Saporin from Saponaria officinalis as a Tool for Experimental Research, Modeling, and Therapy in Neuroscience. *Toxins* 12:546. 10.3390/toxins12090546 32854372 PMC7551693

[B5] Cembrowski M. S. Bachman J. L. Wang L. Sugino K. Shields B. C. Spruston N. (2016). Spatial Gene-Expression Gradients Underlie Prominent Heterogeneity of CA1 Pyramidal Neurons. *Neuron* 89 351–368. 10.1016/j.neuron.2015.12.013 26777276

[B6] Conner J. Franks K. Titterness A. Russell K. Merrill D. Christie B. (2009). NGF is essential for hippocampal plasticity and learning. *J. Neurosci.* 29 10883–10889. 10.1523/JNEUROSCI.2594-09.2009 19726646 PMC2765804

[B7] Cooper J. Salehi A. Delcroix J. Howe C. Belichenko P. Chua-Couzens J. (2001). Failed retrograde transport of NGF in a mouse model of Down’s syndrome: reversal of cholinergic neurodegenerative phenotypes following NGF infusion. *Proc. Natl. Acad. Sci. U S A.* 98 10439–10444. 10.1073/PNAS.181219298 11504920 PMC56979

[B8] Dannenberg H. Young K. Hasselmo M. (2017). Modulation of Hippocampal Circuits by Muscarinic and Nicotinic Receptors. *Front. Neural Circuits* 11:00102. 10.3389/FNCIR.2017.00102 29321728 PMC5733553

[B9] Deacon R. M. J. Rawlins J. N. P. (2006). T-maze alternation in the rodent. *Nat. Protoc.* 1 7–12. 10.1038/nprot.2006.2 17406205

[B10] Dobryakova Y. V. Kasianov A. Zaichenko M. I. Stepanichev M. Y. Chesnokova E. A. Kolosov P. M. (2018). Intracerebroventricular Administration of 192IgG-Saporin Alters Expression of Microglia-Associated Genes in the Dorsal But Not Ventral Hippocampus. *Front. Mol. Neurosci.* 10:429. 10.3389/fnmol.2017.00429 29386992 PMC5776139

[B11] Dobryakova Y. V. Stepanichev M. Y. Markevich V. A. Bolshakov A. P. (2020a). Long-term potentiation in the hippocampal CA3 to CA1 synapses may be induced in vivo by activation of septal cholinergic inputs. *Int. J. Neurosci.* 2020:1822834. 10.1080/00207454.2020.1822834 32916077

[B12] Dobryakova Y. V. Zaichenko M. I. Bolshakov A. P. Stepanichev M. Y. Gulyaeva N. V. Markevic V. A. (2020b). Behavioral effects of immunotoxin 192-IgG-saporin depend on the type of administration. *Zhurnal Vyss. Nervn. Deyatelnosti Im. I.P. Pavlov.* 70 783–793. 10.31857/S0044467720060039

[B13] Dobryakova Y. V. Volobueva M. N. Manolova A. O. Medvedeva T. M. Kvichansky A. A. Gulyaeva N. V. (2019). Cholinergic Deficit Induced by Central Administration of 192IgG-Saporin Is Associated With Activation of Microglia and Cell Loss in the Dorsal Hippocampus of Rats. *Front. Neurosci.* 13:146. 10.3389/fnins.2019.00146 30930730 PMC6424051

[B14] Dobryakova Y. V. Zaichenko M. I. Spivak Y. S. Stepanichev M. Y. Markevich V. A. Bolshakov A. P. (2021). Overexpression of Nerve Growth Factor in the Hippocampus Induces Behavioral Changes in Rats with 192IgG-Saporin-Induced Cholinergic Deficit. *Neurochem. J.* 15 273–281.

[B15] Drachman D. A. Leavitt J. (1974). Human Memory and the Cholinergic System: A Relationship to Aging? *Arch. Neurol.* 30 113–121. 10.1001/archneur.1974.00490320001001 4359364

[B16] Emre M. Aarsland D. Albanese A. Byrne E. J. Deuschl G. De Deyn P. P. (2004). Rivastigmine for Dementia Associated with Parkinson’s Disease. *N. Engl. J. Med.* 351 2509–2518. 10.1056/nejmoa041470 15590953

[B17] Eu W. Z. Chen Y. J. Chen W. T. Wu K. Y. Tsai C. Y. Cheng S. J. (2021). The effect of nerve growth factor on supporting spatial memory depends upon hippocampal cholinergic innervation. *Transl. Psychiatry* 11 1280–1283. 10.1038/s41398-021-01280-3 33723225 PMC7961060

[B18] Fischer W. Björklund A. Chen K. Gage F. (1991). NGF improves spatial memory in aged rodents as a function of age. *J. Neurosci.* 11 1889–1906. 10.1523/JNEUROSCI.11-07-01889.1991 1648601 PMC6575486

[B19] Fletcher B. R. Baxter M. G. Guzowski J. F. Shapiro M. L. Rapp P. R. (2007). Selective cholinergic depletion of the hippocampus spares both behaviorally induced arc transcription and spatial learning and memory. *Hippocampus* 17 227–234. 10.1002/hipo.20261 17286278

[B20] Frielingsdorf H. Simpson D. R. Thal L. J. Pizzo D. P. (2007). Nerve growth factor promotes survival of new neurons in the adult hippocampus. *Neurobiol. Dis.* 26 47–55. 10.1016/j.nbd.2006.11.015 17270453

[B21] Gil-Bea F. J. Solas M. Mateos L. Winblad B. Ramírez M. J. Cedazo-Mínguez A. (2011). Cholinergic hypofunction impairs memory acquisition possibly through hippocampal Arc and BDNF down regulation. *Hippocampus* 21 999–1009. 10.1002/hipo.20812 20865740

[B22] Hefti F. (1994). Neurotrophic factor therapy for nervous system degenerative diseases. *J. Neurobiol.* 25 1418–1435. 10.1002/neu.480251109 7852995

[B23] Hirotsu I. Hori N. Katsuda N. Ishihara T. (1989). Effect of anticholinergic drug on long-term potentiation in rat hippocampal slices. *Brain Res.* 482 194–197. 10.1016/0006-8993(89)90561-1 2706478

[B24] Lee Y.-S. Danandeh A. Baratta J. Lin C.-Y. Yu J. Robertson R. T. (2013). Neurotrophic factors rescue basal forebrain cholinergic neurons and improve performance on a spatial learning test. *Exp. Neurol.* 249 178–186. 10.1016/j.expneurol.2013.08.012 24017996 PMC3939719

[B25] Lehmann O. Jeltsch H. Lazarus C. Tritschler L. Bertrand F. Cassel J. C. (2002). Combined 192 IgG-saporin and 5,7-dihydroxytryptamine lesions in the male rat brain: A neurochemical and behavioral study. *Pharmacol. Biochem. Behav.* 72 899–912. 10.1016/S0091-3057(02)00752-9 12062580

[B26] Lehmann O. Jeltsch H. Lehnardt O. Pain L. Lazarus C. Cassel J. C. (2000). Combined lesions of cholinergic and serotonergic neurons in the rat brain using 192 IgG-saporin and 5,7-dihydroxytryptamine: Neurochemical and behavioural characterization. *Eur. J. Neurosci.* 12 67–79. 10.1046/j.1460-9568.2000.00881.x 10651861

[B27] Loesche J. Steward O. (1977). Behavioral correlates of denervation and reinnervation of the hippocampal formation of the rat: recovery of alternation performance following unilateral entorhinal cortex lesions. *Brain Res. Bull.* 2 31–39. 10.1016/0361-9230(77)90022-3 861769

[B28] Mchugh S. B. Francis A. Mcauley J. D. Stewart A. L. Baxter M. G. Bannerman D. M. (2015). Hippocampal Acetylcholine Depletion Has No Effect on Anxiety, Spatial Novelty Preference, or Differential Reward for Low Rates of Responding (DRL) Performance in Rats. *Behav. Neurosci.* 129 491–501. 10.1037/bne0000072 26214215 PMC4516321

[B29] Motooka Y. Kondoh T. Nomura T. Tamaki N. Tozaki H. Kanno T. (2001). Selective cholinergic denervation inhibits expression of long-term potentiation in the adult but not infant rat hippocampus. *Dev. Brain Res.* 129 119–123. 10.1016/s0165-3806(01)00179-1 11454420

[B30] Nilsson O. G. Leanza G. Rosenblad C. Lappi D. A. Wiley R. G. Bjorklund A. (1992). Spatial learning impairments in rats with selective immunolesion of the forebrain cholinergic system. *Neuroreport* 3 1005–1008. 10.1097/00001756-199211000-00015 1482757

[B31] Pagano G. Rengo G. Pasqualetti G. Femminella G. D. Monzani F. Ferrara N. (2015). Cholinesterase inhibitors for Parkinson’s disease: A systematic review and meta-analysis. *J. Neurol. Neurosurg. Psychiatry* 86 767–773. 10.1136/jnnp-2014-308764 25224676

[B32] Palacios-Filardo J. Udakis M. Brown G. A. Tehan B. G. Congreve M. S. Nathan P. J. (2021). Acetylcholine prioritises direct synaptic inputs from entorhinal cortex to CA1 by differential modulation of feedforward inhibitory circuits. *Nat. Commun.* 121 1–16. 10.1038/s41467-021-25280-5 34531380 PMC8445995

[B33] Paxinos G. Watson C. (2007). *The Rat Brain in Stereotaxic Coordinates.* Amsterdam: Elsevier. 10.1016/0165-0270(80)90021-7 6110810

[B34] Petrella C. Ciotti M. T. Nisticò R. Piccinin S. Calissano P. Capsoni S. (2020). Involvement of Bradykinin Receptor 2 in Nerve Growth Factor Neuroprotective Activity. *Cells* 9:cells9122651. 10.3390/cells9122651 33321704 PMC7763563

[B35] Ramirez J. Caldwell J. Majure M. Wessner D. Klein R. Meyer E. (2003). Adeno-associated virus vector expressing nerve growth factor enhances cholinergic axonal sprouting after cortical injury in rats. *J. Neurosci.* 23 2797–2803. 10.1523/JNEUROSCI.23-07-02797.2003 12684466 PMC6742064

[B36] Ramirez J. McQuilkin M. Carrigan T. MacDonald K. Kelley M. (1996). Progressive entorhinal cortex lesions accelerate hippocampal sprouting and spare spatial memory in rats. *Proc. Natl. Acad. Sci. U. S. A.* 93 15512–15517. 10.1073/PNAS.93.26.15512 8986843 PMC26436

[B37] Reeves T. Smith D. (1987). Reinnervation of the dentate gyrus and recovery of alternation behavior following entorhinal cortex lesions. *Behav. Neurosci.* 101 179–186. 10.1037/0735-7044.101.2.179 3580120

[B38] Schliebs R. Roβner S. Bigl V. (1996). Chapter 25 Immunolesion by 192IgG-saporin of rat basal forebrain cholinergic system: a useful tool to produce cortical cholinergic dysfunction. *Prog. Brain Res.* 109 253–264. 10.1016/S0079-6123(08)62109-3 9009714

[B39] Schulz P. E. Cook E. P. Johnston D. (1995). Using paired-pulse facilitation to probe the mechanisms for long-term potentiation (LTP). *J. Physiol.* 89 3–9. 10.1016/0928-4257(96)80546-8 7581296

[B40] Sokolov M. V. Kleschevnikov A. M. (1995). Atropine suppresses associative LTP in the CA1 region of rat hippocampal slices. *Brain Res.* 672 281–284. 10.1016/0006-8993(94)01376-S 7749748

[B41] Staniszewska I. Sariyer I. K. Lecht S. Brown M. C. Walsh E. M. Tuszynski G. P. (2008). Integrin α9β1 is a receptor for nerve growth factor and other neurotrophins. *J. Cell Sci.* 121 504–513. 10.1242/jcs.000232 18230652 PMC2744358

[B42] Stepanichev M. Markov D. Pasikova N. Gulyaeva N. (2016). Behavior and the cholinergic parameters in olfactory bulbectomized female rodents: Difference between rats and mice. *Behav. Brain Res.* 297 5–14. 10.1016/j.bbr.2015.09.033 26431763

[B43] Sweatt J. (2001). The neuronal MAP kinase cascade: a biochemical signal integration system subserving synaptic plasticity and memory. *J. Neurochem.* 76 1–10. 10.1046/J.1471-4159.2001.00054.X 11145972

[B44] Tasic B. Yao Z. Graybuck L. T. Smith K. A. Nguyen T. N. Bertagnolli D. (2018). Shared and distinct transcriptomic cell types across neocortical areas. *Nature* 563 72–78. 10.1038/s41586-018-0654-5 30382198 PMC6456269

[B45] Volobueva M. N. Dobryakova Y. V. Manolova A. O. Stepanichev M. Y. Kvichansky A. A. Gulyaeva N. V. (2020). Intracerebroventricular Administration of 192IgG-Saporin Alters the State of Microglia in the Neocortex. *Neurochem. J.* 14 37–42. 10.1134/S1819712420010213

[B46] Walsh T. J. Kelly R. M. Dougherty K. D. Stackman R. W. Wiley R. G. Kutscher C. L. (1995). Behavioral and neurobiological alterations induced by the immunotoxin 192-IgG-saporin: cholinergic and non-cholinergic effects following i.c.v. injection. *Brain Res.* 702 233–245. 10.1016/0006-8993(95)01050-X 8846082

[B47] Williams L. R. Varon S. Peterson G. M. Wictorin K. Fischer W. Bjorklund A. (1986). Continuous infusion of nerve growth factor prevents basal forebrain neuronal death after fimbria fornix transection. *Proc. Natl. Acad. Sci. U. S. A.* 83 9231–9235. 10.1073/pnas.83.23.9231 3466184 PMC387109

[B48] Winkler J. Thal L. J. (1995). Effects of nerve growth factor treatment on rats with lesions of the nucleus basalis magnocellularis produced by ibotenic acid, quisqualic acid, and AMPA. *Exp. Neurol.* 136 234–250. 10.1006/exnr.1995.1100 7498413

[B49] Winkler J. Ramirez G. A. Thal L. J. Waite J. J. (2000). Nerve growth factor (NGF) augments cortical and hippocampal cholinergic functioning after p75NGF receptor-mediated deafferentation but impairs inhibitory avoidance and induces fear-related behaviors. *J. Neurosci.* 20 834–844. 10632613 10.1523/JNEUROSCI.20-02-00834.2000 PMC6772410

[B50] Zeisel A. Muñoz-Manchado A. B. Codeluppi S. Lönnerberg P. La Manno G. Juréus A. (2015). Cell types in the mouse cortex and hippocampus revealed by single-cell RNA-seq. *Science* 347 1138L–1142. 10.1126/science.aaa1934 25700174

[B51] Zhang H. Petit G. Gaughwin P. Hansen C. Ranganathan S. Zuo X. (2013). NGF rescues hippocampal cholinergic neuronal markers, restores neurogenesis, and improves the spatial working memory in a mouse model of Huntington’s Disease. *J. Huntingtons. Dis.* 2 69–82. 10.3233/JHD-120026 25063430

